# Progress of Dentistry during Year Ending May 1st, 1889

**Published:** 1889-07

**Authors:** Rodrigues Ottolengui


ARTICLE V.

### PROGRESS OF DENTISTRY DURING YEAR ENDING MAY 1ST, 1889.

REPORT OF COMMITTEE ON PRACTICE TO THE DENTAL SOCIETY OF THE STATE OF NEW YORK.

BY RODRIGUES OTTOLENGUI.

*Mr. President and Gentlemen.*—Your committee has decided to offer you a report covering many branches in our profession, and therefore will proceed without any preamble, to be as brief as possible.

*Surgery.*—The operation of implanting teeth has attracted more attention than any new feature of dental progress in the last decade. There has been abundant evidence to prove that it is possible for a tooth so placed to become firmly attached to the jaw. What the exact nature of this attachment it has not, in the opinion of your committee, been thoroughly settled. Dr. G. L. Curtiss's evidence in favor of anchylosis must stand until further investigation proves him wrong. As yet no sections have been through an implanted tooth and surrounding alveolus. Several gentlemen, however, are working in this direction, conspicuous among the number being Dr. Sudduth. We may therefore expect more data at an early date. Meanwhile an important point seems to have been settled which bears on the ethics of performing this operation. The teeth in their normal sockets are held by an intervening soft tissue—the peri cementum—between the tooth root and the bone. This wise provision of nature allows severe shocks to be received without injury. The force with which the jaws are brought together is greater than commonly supposed, as one discovers when the tongue is accidently caught in chewing. The implanted tooth, however attached, is fixed. There is no protecting resiliency. It may therefore be retained indefinitely, and then rapidly lost by a retrograde process, the attachment first being shattered and rapid absorption of the root resulting. Your committee must not be misunderstood as indicating that there is no absorption of the implanted tooth in the first instance, the bays being filled in as is claimed by some. That is not yet determined. The point wished to be made prominent is the fact that when absorption sets in, *after the tooth has once become firm*, the destructive process is likely to be very rapid. The question arises, can such a condition be restored to health? Your committee has been most interested in this problem. During the year two such cases were treated. In both instances the implanted teeth had been in place over eighteen months. In one the fistula was healed after new bone had been induced to form, and to-day, five months later, all is well. In the second case, after the most strenuous efforts, including an attempt at reproduction of tissue by sponge-grafting the tooth was lost. That one case responded to treatment, however, is the point of value, since it proves that under favoring circumstances such healing is *possible*.

Such great progress has been made in crown and bridge work, and the success of implanting teeth is still so doubtful, that your committee would advise that implantation be resorted to *only where all other methods would prove failures*.

*Pyorrhea Alveolaris.*—This disease is one which is at this time receiving renewed attention in consequence of the report of your last year's committee to the effect that in many instances the most unpromising cases yield readily enough if the treatment is conscientiously followed up by both dentist and patient. Your committee for this year has to report that a number of cases have been treated and seen during the year, where the method of bridging together at the cutting edges with gold had been resorted to, and the results were most gratifying. This line of treatment may be thoroughly understood by any not already acquainted with it, by referring to articles in the journals during the last year, or by corresponding directly with Dr. M. L Rhein.

*Alveolar Abscess.*—The advocates of immediate root filling have been so loud in their hurrahing and so convincing in their arguments that they have converted the majority of our profession to their method. This, however, leaves alveolar abscess to be considered from a new standpoint. If the root is to be filled at the first sitting, it is possible in many cases where the condition of the end of the root is obscure, that shortly after such treatment positive evidences of abscess may present. To those accustomed to treat such conditions through the root canal this is a point to be pondered over. In such instances your committee has to report that the most satisfactory result may be hoped for, if the sac be burred off the end of the root with a sharp bur, passed through the soft parts and the alveolar wall. Where such an operation seems likely to produce pain, cocaine or gas may be administered, or ether spray may be used. Where pain may result and continues afterward, it may be abated with five grain doses of antipyrine. In performing this burring operation on superior bicuspids or the buccal roots of superior molars, care should be taken not to enter the antrum, even though such an accident would not of necessity be attended by untoward consequences.

*Drilling through Roots.*—Your committee during the year has seen teeth lost in four cases by this accident, followed by unwise after treatment. In one a screw for a crown was passed through the side of a root, by actual measurement three-sixteenths of an inch, entering the process that far. A sequestrum of bone one inch long resulted from necrosis and four teeth were lost. In a second case a double rooted bicuspid was mistaken for a single rooted tooth, and in trying to form a straight canal the drill was passed between the two roots; gutta percha was passed into this hole, and the tooth filled with amalgam. Subsequently a great abscess formed and the patient lost confidence in the dentist. Every known remedy was tried, the roots being burred, but after three weeks' endeavor the tooth was removed. As this accident was at the hands of one of our most prominent members it would seem that your committee is right in recommending extra caution in drilling into root canals. The other two cases, both molars, were similar, and both were lost. Still a fifth case, a molar, however, was saved. In that case the bur had passed through near the tooth neck, and no abscess was present. Decay had naturally attacked the tooth at that point, and a large cavity was found under the gum. The tooth was saved by thorough filling. It might be mentioned in passing that in the case of the bicuspid, another was implanted in its place, and seen within a month, six months later, is doing well. In that case the soft tissues were trimmed away about the hypertrophied borders of the fistulous opening with the scissors, and painted freely with salicylic acid (sat. sol. alcohol).

*Filling Materials and Methods.*—In this connection your committee would call attention to various points.

*Porcelain.*—Porcelain as a filling material seems to be a failure. In small cavities it cannot be used. In Proximal cavities almost invariably, granting that a perfect filling can be baked, it is necessary to make unwarrantable destruction of tooth material in order that these fillings may be forced into position after they are ready. This might be excused, other objections being done away with, if the sacrifice was at the palatal aspect. In most of the cases exhibited to your committee, however, the reverse has been the practice, and the operators have cut away the labial faces of teeth, trusting to their skill to restore with porcelain. Your committee claims, however, that thus far fillings have not been made possible, either in regard to exactness of adaptation or of color. Tooth body, as is well known, is baked at a temperature which cannot be measured. The color depends to a very great degree on temperature, therefore it is manifestly impossible to obtain a given color, even though exact proportions of bodies be used together. As this is admitted by the manufacturers of teeth, it seems absurd for a dentist to hope for better results. As to adaptation, the amount of shrinkage depends on varying causes, and therefore is not controllable. Space exists at the margins, admittedly the most important part of any filling, and these are occupied by phosphates. Thus what should be the strongest point, in this kind of work is the weakest. When we can produce a phosphate or other light colored plastic which will not dissolve in the fluids of the mouth, we will be able to make porcelain fillings at least permanent. But when we have attained that goal we will not need porcelain at all, because we will be able in those halcyon days to produce better results with the plastic alone.

*Copper Amalgam.*—This material undoubtedly will continue to hold the high place in our confidence which it does at present. There are many places where it will serve us better than anything else. As to color, that is a question of æsthetics, and it is only a matter of opinion which is more beautiful; ivory or ebony. Copper amalgam, if properly used and conscientiously polished at a subsequent sitting (the lapse of a week is better than a shorter time) will give us a surface like polished ebony, and we may educate ourselves and our patients to admire these beautiful black fillings, especially since the edges are so perfect. There is however, a fact to be remembered always, and that is, that copper amalgam is not easily removed with a drill or bur. If a pulp is nearly exposed in an obscure cavity, say posterior proximal of second molar below, it will be safer after capping not to fill with copper amalgam, for should the tooth ache subsequently and it became necessary to remove the filling to reach the pulp great difficulty will present.

*Plastic Golds.*—The preparations known by this name may be made very serviceable provided they be not used exclusively. Either with crystal, or crystaloid gold, undoubtedly good fillings may be made in some instances, but such work is surely no better than the ordinary gold filling in any case, whereas in many instances it would prove entirely unsatisfactory. Therefore the use of these materials for entire fillings is to be deprecated. In sensitive teeth, however, where the cavity can be less painlessly made simply of a general retaining shape, without grooves or retaining pits, the plastic gold is invaluable for starting the filling, and may be used with safety for one-half or or even two-thirds of the depth of the cavity, the completion being with foil, preferably of heavy gauge.

*Gold aud Iridium.*—This is a preparation suggested by Dr. Wheeler of Albany. Iridium is laid between layers of gold, and rolled down to the thickness of forty or sixty foil. It makes a light colored filling with a glassy surface, which will wear better than any combination of gold in exposed grinding surfaces. It is, however, very difficult to manipulate, being very stiff and unyielding. Nevertheless it has a place among our filling materials, and when needed, however rarely, there is scarcely any substitute so good.

*Gold and Platinum.*—This is a combination which makes a very dense, durable filling, of light color. It is specially indicated for extensive contours in conspicuous positions, or where great strength is required, as in uniting teeth for pyorrhea. Those who have never used it should be very cautious at first, as this material is exceedingly difficult to manipulate. It tears readily under small points, and nevertheless too large foot pluggers should not be used. A long narrow foot plugger with fine serration seems best. The main point, however, is annealing. It is necessary, to obtain best results, that this should be thoroughly done and yet there is danger of burning off the thin outer layer of gold, exposing the platinum and thus preventing cohesion. Therefore it will be wiser not to depend on skill, but to use the Johnson annealing apparatus with which the material may be heated to a bright red without burning. This devicc may be had of S. S. White & Co. It is in appearance like a small nutmeg grater, set at an incline over the spirit lamp, the heat passing through the little holes to reach the gold which is laid on the upper side. It is safer to anneal a large piece and cut in strips afterward.

*Gold and Amalgam.*—It has been demonstrated again during the year, though long ago discovered by Dr. Kingsley, that gold may be made to unite with amalgam at the same sitting, thus giving amalgam as a protection to the cervical border, where it has proven better than gold, and gold along all other edges where it is evidently better than most amalgams. In filling proximal cavities after this method a matrix is needed, securely placed. Amalgam is packed along the cervical border, and immediately upon it is packed plastic gold, preferably Steurer's until the gold color persists, when the filling may be finished as any other gold filling with any foil which the operator may prefer. Your committee was showed test fillings made in this manner, a tube of glass one inch long having been filled half with amalgam and half with gold. Removed from the tube the union between the two was so perfect that it was impossible to break them apart with the fingers. Your committee recommends this method in cases where from extensive decay below the gum margin it would be difficult or impossible to fill with gold. When the depredation is so great that the cervical edge cannot be reached with the matrix, that portion may be filled before placing the dam in position, and the matrix adjusted afterward, when more amalgam must be put over that first inserted, to insure a surface free from moisture, before adding the plastic gold.

*Gold and Oxy-phosphate.*—Your committee wishes to attract special attention to a method devised by Dr. Reese of Brooklyn. In shallow cavities, the pulp being not exposed but nearly so, and where it would be dangerous to obtain sufficient retaining shape to the cavity, some "sticky" grade of oxy-phosphate may be placed over the bottom of the cavity,and before it is allowed to set a pellet of gold large enough to fill the cavity, is pressed gently into the cement, and then left undisturbed. When the cement has set it will be found that the piece of gold is firmly held; it may then be condensed and forms the foundation on which the filling is built. In this way the filling is actually *cemented into position.* Your committee after trial of more than two years has no hesitation in saying that this is a thoroughly reliable method of starting a filling and of additionally securing it. Of course it is not meant that large contours may be made to depend on the cement alone; judgement must be used, but a trial will convince anyone that this is a most valuable idea. It also serves well with amalgam fillings, possibly better since such a filling may be completed without waiting for the cement to set.

*Robinson's felt.*—This material is excellent for children's teeth and for beginning large cup shaped crown cavities, or any others of great depth. It is claimed that gold coheres to it. This in the opinion of your committee, is an error. The felt may be placed in the lower part of a cavity and gold built on it. But the union is mechanical; that is, deeply serrated instruments must be used, thus forcing the gold into the softer mass of felt; in this way it serves well, clutching the gold sufficiently to aid us in making the filling. This union, however, must be depended on no further, and the cavity after it is partly filled with the felt must still offer a shape which will retain a filling. Your committee has looked into this thoroughly, and after long-continued use sufficient to acquire needed dexterity in manipulating the material asserts that if a tube were filled half with felt and half with gold, there would be little difficulty to separate them. As, however, the union is sufficient for working purposes, there are many cases where a great deal of time may be saved the operator and fatigue to the patient, if one-half or two thirds of a cavity be filled with the felt. As it seems to contain asbestos (?) it is an excellent material to underlie either gold or amalgam where the pulp has been nearly approached by the caries.

*Amalgam.*—In spite of all that the gold adherents, and copper amalgam advocates may say, there remains still a vast number of teeth where a light colored serviceable amalgam is loudly called for. Immense proximal cavities in bicuspid teeth will continue to occur in the mouths of those who cannot afford a large fee for a crown or contour in gold. Copper amalgam from its color is not to be thought of. A method then of filling such cavities in a durable way so that the patient may afford the fee and the dentist the time is an important need. Such a method was advocated by your last committee, and has probably been but little appreciated or believed. Substantially it is as follows. The dam is to be placed, though that should precede every filling of every kind whenever possible. An amalgam known to be reliable, especially as to color, is mixed rather dry and the tooth filled. Very thin gold foil, thinner than what is known as No. 4, is annealed and cut into small strips, which are laid unfolded on the filling, and with perfectly clean warm burnishers (which should be kept specially for this purpose) burnished into the filling until it has set hard. It must be done thoroughly and sufficient gold used; sometimes a sheet or even two sheets will be required. The result is an ideal filling which will keep its color and its finish. Your present committee not only has tested this in practice during the year, but was allowed to inspect fillings made in this manner by the chairman of last year, where they had been in position two, three and four years. These fillings are so beautiful that those who see them for the first time involuntarily ask, "What amalgam is that?"

*Treatment of Exposed Pulps.*—This is a delicate subject because of the fact that there are many, high in the profession, of whose friendship your committee is proud, with whom, however, is necessary to differ. A decade ago, or about that time, a movement in favor of pulp capping swept over our profession and took such a strong hold on our fellows that scarce one could be found with temerity to say a word contrary to the new method. What is worse, our young men were taught that almost any pulp which would bleed could be saved. So many failures, nevertheless, occured from time to time, that unwilling to abandon the theory, men sought a remedy in new materials, till to-day our records would show that pulps have been capped with everything from tar to tooth-picks. Your committee could readily give forty or fifty different cappings. It seems time that some public word should be spoken aloud by an authoritative body to discountenance this, and to encourage those who, even now, have secretedly abandoned the practice, to come forward openly and tell us what they are doing. We need a record of practice, not of theories. Ten years ago or more, when the death of a pulp meant the probable loss of the tooth, it was expedient to take every risk to save that little life, but to-day when the march of antisepsis has placed the wide-awake dentist almost beyond the danger of alveolar abscess, in the opinion of your committee it is almost as ill-advised to cap as it was then to kill. Your committee wishes it understood that the possibility of saving exposed pulps is not denied, even where amputation of a part has been resorted to ; it is known that such success is possible—nay, even that the chances would altogether favor such an outcome *where the patient is in good general health.* But just there is the difficulty. Many exposed pulps are brought to us for treatment by persons with very ill-nourished bodies. Therefore, admitting the possibilities, your committee still decides against capping pulps, because the statistics undoubetdly show more failures than successes. If the exposed pulp be removed, and in the light of the antiseptic teaching of today the tooth-root be hygienically purified and filled, that tooth is safer than where a gold filling overlies a capping, giving the patient a fancied security which will not be disturbed, till in the dark hours of some night he is awakened to the truth with a fine specimen of an abscess as a companion. Your committee, however, deems it nearly always safe to cap *where the pulp has not been actually exposed.* In such cases where the tooth has ached a mild dressing of equal parts of creosote and oil of cloves should be exhibited for twenty-four hours before capping, and a temporary gutta percha filling be placed for several days before final filling. If after such treatment the pulp gives no warning throbs, it would seem safe to fill permanently.

*Root Filling.*—The immediate root filling advocates have covered themselves with glory and are masters of the field. So far as abscess after filling is concerned, that has been already touched on in this report. Your committee has but to outline what seems the best method of filling the root. Perhaps the most commonly used filling material is gutta percha, and next in order is oxy-phosphate. Neither of these materials is as reliable as the profession think. At a meeting in Philadelphia this winter a gentleman exhibited the results of some experiments in this direction which were both instructive and important. He filled roots out of the mouth with gutta percha, and others with oxy-phosphate. Twenty four hours later they were placed in aniline ink and left for one day. A segment-shaped section was removed and it was discovered that the ink had entered the dentine from the outside through the cementum, and from the inside by way of the canal, despite the fillings, which are generally thought to be water proof. A third specimen was where the root canal had been filled with cotton as a vehicle to carry caıbolized cosmoline. The cosmoline had permeated the tubuli and thus prevented the ingress of the ink either from the outer surface or from within. Your committee does not think it advisable to use cotton as a root filling; nevertheless the above experiments teach the value of allowing a greasy filling to enter and carry with it a disinfectant. It therefore seems that the very best method is that introduced by Dr. Van Woert, which is to half fill the canal with a paste made of iodol, zinc oxide, and carbolized vaseline. By mixing these a paste cau be formed stiff enough to render cotton unnecessary in its introduction. The upper half should be filled with oxy-phosphate, or better still oxy-chloride of zinc. In practice this has proven as good as it looks theoretically considered. All manner of teeth have been treated in this way, and the percentage of success is most encouraging, A great many men in New York and Brooklyn, in Boston and Philadelphia, have adopted this, and all report favorably.

*Crowns.*—This is a subject on which a book might be written; in fact, a book has been written and is already, with a year, in its second edition. Your committee wishes only to call attention to one or two comparatively new methods.

What is known as the *porcelain cusp crown*, is now offered by one of our manufacturers, and is an excellent method easily applied to many cases of molars and bicuspids. A band of gold is fitted about the root and the rubber dam is then applied. This is perhaps the only method by which we can be assured that the cement used is not reached by moisture. The dam in place, the adaptation of the band may be made accurate; the cement is introduced, and the cusp of porcelain which has one pin is then pressed into place, forming a most admirable crown.

*All Gold Crowns.*—All are familiar with the various methods of joining a cusp which has been swaged, to a band, producing an all gold crown. Although ready made dies and die plates may be had, the best results by this method are only had when special dies are made for each case. Even then, after great care, it will often occur that the crown is too long and it cannot be ground, as that would produce a hole through the cusp in many instances. Your committee therefore, where the "bite" is short, suggests the following method: After the band is fitted to the root, it is trimmed to mark the length of the bite; that is, it is made to occlude with the opposing tooth. Thin platinum is then burnished over the end of the root within the band, which is in place, and waxed to the band. This is carefully removed, invested, and loaded up with gold plate. The upper part being solid may readily be ground to make the occlusion accurate, and cusps formed with engine-burs. When finished it is all gold, no solder having been used except to unite the ends of the band (and that should be 20 carat), and thus a good color is secured.

*Gold Tips.*—A new method of making gold tips for abraded teeth has been introduced to the profession during the year by Dr. Van Woert. As this report is already long, however, it will be as well to say here that it is a most valuable addition to our resources, and that a full description of the method and instruments may be found in the *Cosmos*. The instruments are now in the market.

*Bridgework.*—This system of restoring lost teeth has come to stay. It, however, is still in its infancy, though a promising child and well worthy of being wedded to the dentist if he can only win her from her present parents, or rather "legal guardians." The weak point about bridgework is its strength. This is a paradox, but it is explainable. If a bridge be united to several roots, it becomes a most difficult matter to remove it. Such removal often involves the total destruction of the bridge. And such removal becomes necessary with our present methods whenever an accident occurs and one tooth is broken. If one tooth is broken on a gold plate it may be repaired at a trifling cost to the patient. Not so the bridge. If codes of ethics mean anything, then it is our duty to devise promptly some reliable system whereby bridges may be so made that repairs may be made inexpensively. It is not just to exact a large fee for a contrivance so made that the patient is constantly in danger of losing its use or paying another large fee. Your committee was shown during the year a tooth invented by Dr. Wheeler, of Albany, which aims at remedying this trouble, and your committee recommends that a committee be appointed to examine into the merits of Dr. Wheeler's tooth, and if said committee report satisfactorily, that this Society recommend our manufacturers to arrange with Dr. Wheeler for the production of such teeth. A similar object is aimed at in a tooth invented by Dr. Perry, of New York. Your committee makes the same recommendation in regard to it.

*Dentures.*– In artificial dentures there are two comparatively new methods.

*Aluminum.*—This material has been shown together with the method of casting it, many times during the year. Your committee has met it at all the large meetings, and everywhere found dentists who had used it speakig in the highest terms of it. Undoubtedly, with good manipulation excellent dentures may be produced in this way. A special excellence is its use in regulating plates; points of attachment for ligatures, and places of resistance for jack-screws can be readily made without occupying much space, and screw threads may be cut through the plate itself through which screws may be made to act excellently well. Your committee has nothing but commendation to offer for this process.

*Deposit Gold Plates.*—Despite the fact that this method has been largely defended lately through the journals, it is the opinion of your committee that unless great improvements be made in this plate it will serve us but slightly. The advantage is that absolute adaptation is secured. The disadvantages are that the plates in many mouths discolor, and that every time any part is filed (to relieve some point of irritation, for example), the plate must be returned to the makers to be replated, as the silver becomes exposed. The plates cannot be soldered, that is they could not be as made a few months ago when your committee examined into the process. Where the teeth are attached with rubber the roughening of the surface as relied on is not sufficient to hold the rubber. Your committee expresses this opinion, although no such accident as the separation of rubber from these plates is known. Your committee, however, has learned by experience with gold plates, that a deeply graved surface of metal, and even holes punched through the plate is often inadequate, especially with pink rubber. Holes cannot be punched through the deposit plate as that would bring the rubber in contact with silver. Your committee in making this adverse criticism is not actuated by any but the friendliest feelings towards the company which makes these plates. Your committee has been well treated by them. These objections, however, are pointed out that the company may be spurred on to overcoming them. The principle of making a plate by depositing metal is one which promises a great deal, and it is to be hoped that by next year a report may be made that the process has been perfected and all obstacles overcome.

Your committee wished to say something of therapeutics, but fears to make this report tediously long. It cannot refrain, however, from predicting that the time must come when the dentist will be a thorough physician, able to prescribe internal as well as to apply topical agents. It is to be hoped that the time is near, and that the knowledge of medicine which will be necessary to the proper practice of our profession will be offered by our colleges, and that a dental graduate will be the peer of the medical school man. Your committee has to report that this prophecy for the future is founded on a demand for such higher education which has been voiced by the leading societies all over the country. The colleges have already granted something and more is promised. It is "ask and have."

Your committee begs leave to submit its report.

—*Brooklyn Medical Journal.*

